# Transposons take remote control

**DOI:** 10.7554/eLife.40921

**Published:** 2018-09-26

**Authors:** Julius Judd, Cédric Feschotte

**Affiliations:** Department of Molecular Biology & GeneticsCornell UniversityIthacaUnited States

**Keywords:** endogenous retroviruses, transposable elements, CRISPR/Cas9, transcriptional regulation, enhancers, gene expression, Human

## Abstract

A family of retroviral-like elements in the human genome has a pervasive influence on gene expression.

**Related research article** Fuentes DR, Swigut T, Wysocka J. 2018. Systematic perturbation of retroviral LTRs reveals widespread long-range effects on human gene regulation. *eLife* **7**:e35989. doi: 10.7554/eLife.35989

Transcriptional enhancers are regions of DNA to which regulatory proteins can bind in order to increase the transcription – and hence expression – of a particular gene. The enhancers form a dense network that acts at many genomic locations. As a result, even subtle changes to the cocktail of regulatory proteins can produce massive changes in transcription.

But where do enhancers come from? How do sequences that respond to the same regulatory proteins become associated with genes scattered across different chromosomes? A typical mammalian genome contains hundreds of thousands of potential enhancers, but the majority are unique to the species they are found in (Long et al., 2016). So what mechanisms drive their repeated emergence?

Barbara McClintock provided evidence of a potent mechanism in her seminal discovery of what she presciently dubbed ‘controlling elements’ – sequences of DNA that can move across the genome. Building on this, in the late 1960s Roy Britten and Eric Davidson proposed a model in which these elements – subsequently renamed transposons – could provide the raw material for complex regulatory networks (Britten and Davidson, 1969).

Evidence in support of the Britten–Davidson model has grown steadily over the last decade (reviewed in Chuong et al., 2017). First, numerous examples of regulatory sequences derived from individual transposons have been documented in a variety of organisms. Furthermore, genomics has made it apparent that distinct suites of regulatory proteins bind to different transposon families. This binding allows groups of transposons to be activated en masse in certain cell types and during certain developmental stages. Now, in eLife, Daniel Fuentes, Tomek Swigut and Joanna Wysocka of Stanford University report that simultaneous perturbation of a family of retroviral-like transposons called LTR5HS produces profound transcriptional changes in human embryonic-like cells (Fuentes et al., 2018). These findings provide the strongest evidence thus far in support of the Britten–Davidson model as a genome-wide paradigm.

While CRISPR/Cas9 is often used for genome editing, the inactive Cas9 enzyme can also work with specific guide RNAs to tether protein domains to a precise location in the genome. The Wysocka lab recently developed a method called CARGO (chimeric array of guide RNA oligonucleotides) that can deliver tens of guide RNAs to a cell, allowing multiple locations to be targeted (Gu et al., 2018). Fuentes et al. have now exploited the CARGO system to activate or repress LTR5HS elements en masse in cultured cells that behave like human embryonic stem cells.

About 15 million years ago the family of retroviruses that gave rise to LTR5HS spread in the germline of ancestral hominids. Because this family has expanded recently in the genome, all LTR5HS elements are very similar in sequence. As a result, Fuentes et al. were able to target around 90% of all the elements with only 12 guide RNAs.

Fuentes et al. coupled this CARGO array with Cas9 fused to protein domains that either activate or inhibit transcription. In response, 275 human genes were reciprocally up- or down-regulated (Figure 1). These genes were often located relatively far from the nearest LTR5HS element, suggesting that the elements acted as transcriptional enhancers. Further support came from looking at chromatin – the structure formed by DNA and proteins to package the DNA into cells. Fuentes et al. show that activating LTR5HS elements causes both the elements and their target genes to acquire marks that open up chromatin – meaning that they can be transcribed more easily. This is despite there being no detectable binding of Cas9 to the target genes. Furthermore, repression of LTR5HS elements leads to repressive chromatin at the elements themselves, but not at the genes they appear to regulate.

**Figure 1.**
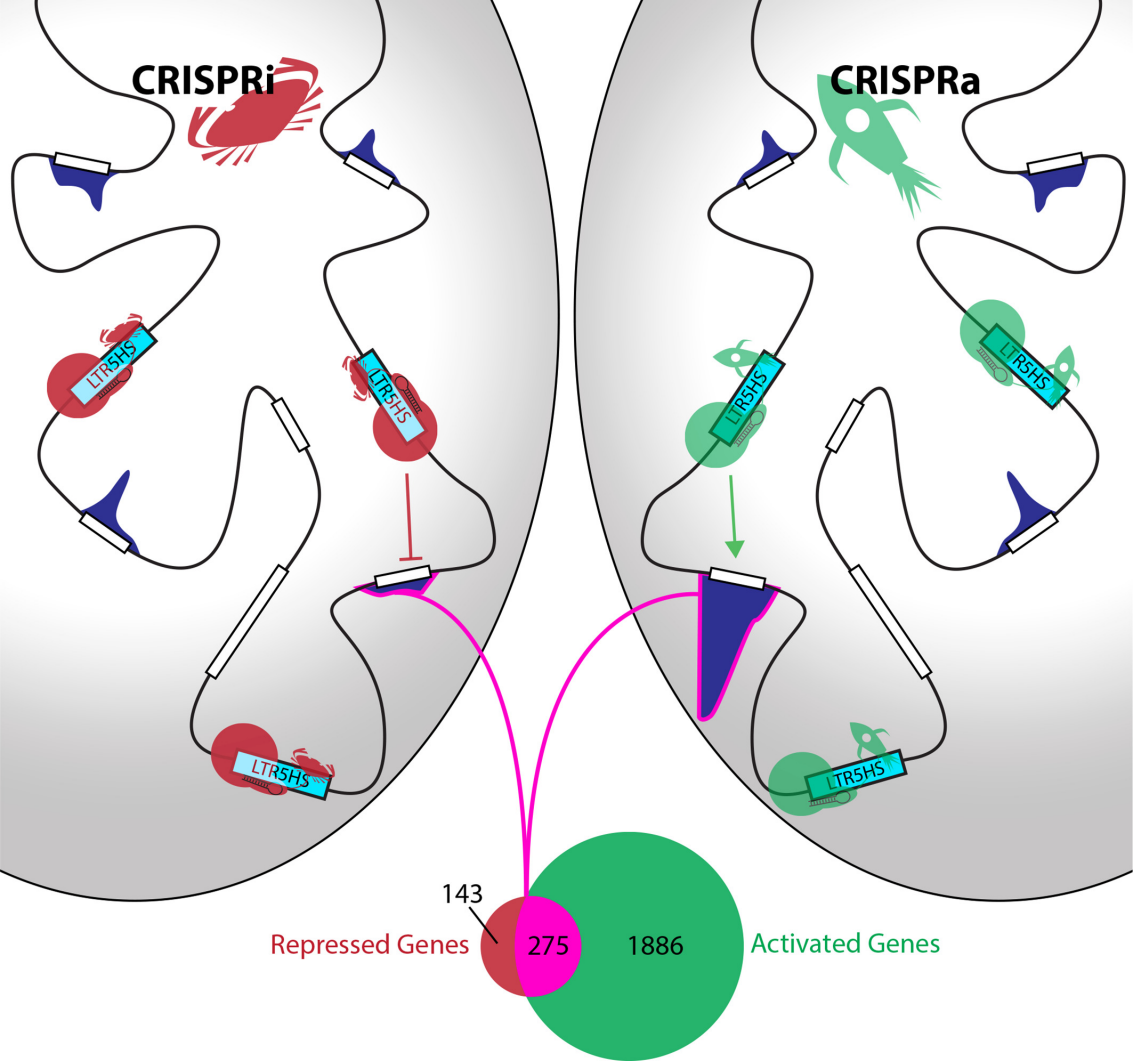
Identification of genes controlled by LRT5HS elements. Fuentes et al. used inactive Cas9 (red or green circles), guided by short RNAs (black hairpins), to deliver either repressive proteins (red crabs) to silence LTR5HS elements (left; CRISPRi), or activating proteins (green rockets) to activate LTR5HS elements (right; CRISPRa). Silencing the elements (represented by cyan boxes) repressed the expression of remote genes (white boxes), while activating the elements enhanced the expression of these genes: the level of expression associated with each gene is shown by a dark blue histogram, and the genome is represented by the black line. The Venn diagram shows that 275 genes (highlighted in pink) are both repressed by CRISPRi and activated by CRISPRa, while another 143 are repressed but not activated, and another 1886 are activated but not repressed.

Fuentes et al. also validate their observations for six separate genes by using CRISPR/Cas9 to delete individual LTR5HS elements. In each case, the deletions led to a significant decrease in the expression of a nearby gene. This is particularly striking because multiple enhancers often act redundantly on the same gene (Osterwalder et al., 2018).

Together, the results of Fuentes et al. suggest that in human embryonic-like cells, a potentially large subset of LTR5HS elements work as enhancers to control the activity of remote genes. However, it remains to be seen whether any of these regulatory activities have provided adaptive benefits during primate evolution. Intriguingly, many of the LTR5HS elements with enhancer activity are human-specific and some are not even fixed in the human population (Wildschutte et al., 2016). This raises the possibility that they contributed to recent adaptations. With CARGO in hand, the answers to these and other outstanding questions shall be delivered.
